# How are the Spiritual Resources and Needs of Mental Health Consumers Identified and Documented by Staff upon Admission to an Australian Mental Health Service? A Mixed Methods Study

**DOI:** 10.1007/s10943-024-02237-8

**Published:** 2025-01-17

**Authors:** Kate Fiona Jones, Megan C. Best

**Affiliations:** 1 Institute for Ethics and Society, University of Notre Dame, PO Box 944, Broadway, NSW 2007 Australia; 2 St Vincent’s Health Network, Sydney, NSW Australia

**Keywords:** Mental health, Healthcare professionals, Spirituality, Religion, Assessment

## Abstract

This study investigated how the spiritual resources and needs of Australian mental health consumers are identified by staff during admission at an inner-city acute care hospital. A mixed-methods study was conducted incorporating an audit of medical records (*n* = 205), and a staff focus group (*n* = 6). The results revealed that information collected during admission is often limited to factors such as medical and social history. Although participants could identify benefits of asking about spirituality, reasons for not asking were also articulated. Staff training and better care planning were identified as two ways to improve awareness of patients’ spiritual needs.

## Introduction

Spirituality has been defined as *“a dynamic and intrinsic aspect of humanity through which persons seek ultimate meaning, purpose, and transcendence, and experience relationship to self, family, others, community, society, nature, and the significant or sacred”* (Puchalski et al., 2014, p. 5). While such a definition may include religious belief, it encompasses other sources of connection and ultimate meaning that may be important for an individual. It has been identified as a key facet of healthcare (World Health Organisation, 1985), and recognised to contribute to the mental health (MH) of healthcare recipients by providing a sense of purpose and meaning (NSW Ministry of Health, 2020), contributing to the healing journey (NSW Ministry of Health, 2020) and fostering greater levels of empowerment (Hipolito et al., 2014).

According to the Australian and New Zealand College of Psychiatrists (RANZCP) (2018) *“religious/spiritual beliefs, values and practices of the individual, their families and of their communities, have the potential to influence the course of mental illness and attitudes towards people living with mental illness”.* Exploration of religion/spiritual issues are therefore of core relevance to the expression and treatment of mental illness. In Australia, government initiatives promoting MH (Australian Government, 2018; National Mental Health Commission, 2022), cultural safety (NSW Ministry of Health, 2020), and trauma-informed care (NSW Government, 2022) have all highlighted the important role spirituality plays in relation to social and emotional well-being.

Research findings relating to the role of spirituality in healthcare have supported policy directives. A recent rapid review found that religion can support MH among Culturally And Linguistically Diverse (CALD) people, and religious or spiritual practices can be adopted as effective MH strategies, with religious leaders identified as important sources of support (Malviya, 2023). Moreover, spirituality was identified as a protective factor, reducing the likelihood or severity of hopelessness, suicidality and depression (Malviya, 2023). This protective aspect of spirituality or religion within the context of mental illness has been identified among a range of populations, such as healthcare professionals during the COVID-19 global pandemic, CALD groups, rehabilitation patients and university students (Jones et al., 2020a, 2020b, 2020c; Koenig et al., 2024; Malviya, 2023; Metry et al., 2024; Pečečnik & Gostečnik, 2022; Wilson et al., 2017). It should be noted that although spirituality is generally associated with positive health outcomes, in some cases spiritual or religious themes have been observed to have a negative impact upon mental health and well-being (Bockrath et al., 2022; Lucchetti et al., 2021; Pargament & Exline, 2022), or been incorporated into an individual’s mental illness (American Psychiatric Association, 2013; Machado & Moreira-Almeida, 2021; Peteet & Lu, 2022). Pargament and Exline (2022) suggest that times of spiritual struggle may result in a person experiencing either a period of decline in their mental health, or growth. Likewise, in a review of the literature, Lucchetti et al. (2021) observed that spirituality or religiousness may have both positive or negative influence upon how individuals cope with distress, and that for these reasons healthcare practitioners should enquire about spiritual or religious beliefs to ensure that good care is provided.

The association between spirituality and positive outcomes align with perspectives of MH consumers (MHCs) themselves (Currier et al., 2020; Yamada et al., 2020), with one study of over 2000 MHCs reporting that over 80% thought spirituality was important to their MH (Yamada et al., 2020). Spiritual practices identified to be helpful included prayer, meditation, attending religious services, reading sacred texts or spiritual self-help books or spending time in nature (Yamada et al., 2020). These findings are similar to those of patients across wider healthcare settings (Best et al., 2022, 2024a, 2024b; Jones et al., 2024a, 2024b; McCrindle & Renton, 2021).

Exploration of the views of MH practitioners (MHPs) has also been undertaken (Neathery et al., 2020; Wade & House, 2022). In one qualitative study (Wade & House, 2022), participants expressed the belief that the spiritual needs of older people with MH problems could be largely met by providing good person-centred care. However, there were conceptual misunderstandings among staff about what spirituality is, with some reluctant to integrate it specifically into their practice. A recent study (Best et al., 2024b) found that only a very small proportion of MHCs (1 out of a total of 6 MHCs) were seen by chaplains, suggesting that staff awareness of the availability of spiritual care specialists may not be high. A small chaplain to patient ratio may also be a contributing factor (Burge, 2024). In comparison, another study identified that participants who identified as “spiritual or religious” or had more years of experience working in MH were more likely to provide spiritual care to patients and reported higher levels of spiritual perspectives (Neathery et al., 2020).

There has been little investigation regarding how the spiritual resources and needs of consumers accessing MH services are documented in their medical record, other than by spiritual care specialists themselves (Goh et al., 2014). The aim of this study was to investigate how the spiritual resources and needs of MHCs are currently identified and documented by medical staff at admission to a MH service.

## Methods

A mixed-methods approach to this study was adopted (Cresswell, 2009), utilising an explanatory sequential design (Ivankova et al., 2006). Such an approach enabled the quantitative findings of the study to be further explored and interpreted through qualitative exploration. This study comprised two components: an audit of medical record documentation, followed by a focus group with MHPs. Ethical approval was granted for both components of the project by the University of Notre Dame Australia Human Research Ethics Committee (reference 2023-081) and the St Vincent’s Hospital Sydney Human Research Ethics Committee (reference 2023/ETH01004).

### Audit

The first component of the project involved an audit of the medical records of 200 consumers admitted to MH services at St Vincent’s Hospital Sydney, an acute inner-city hospital. The five services included were: (i) the safe assessment space in the emergency department (SAS); (ii) psychiatric emergency care centre (PECC); (iii) inpatient mental health care (IMH); iv) community mental health (CMH); and v) older people’s mental health (OPMH). An equal number of medical records from each service was randomly selected by the Health Information Service at the hospital. The medical records of MHCs were included if they had been admitted to the service on or after the 1 September 2022, and the admission had been for at least three days duration. Written consent was not obtained from the patients whose medical records were audited and deemed unnecessary by the Ethics Committees. Many patient details could have changed since discharge, and therefore contacting them may have been difficult. All demographic data was collated, and staff documentation was de-identified.

Documentation by healthcare professionals in the medical records during the first three days (inpatient records) or first three interventions (community records) of the admission was reviewed for information pertaining to the spiritual resources or needs of the MHC. Entries in the medical records were examined for any mention of key words which may be associated with spiritual strengths or need, such as (but not limited to) ‘spirituality’, ‘religion’, ‘meaning’, ‘culture’, ‘purpose in life’, ‘growth’, ‘hope’, ‘faith’, or ‘belief’. Any raw data including such terms was included in the analysis.

A coding strategy was adopted to organise the file data into four categories, dependent on what information was included in the initial documentation and assessments by staff:

1. No documentation about spiritual resources or needs (medical or social information only).
2. Some documentation about resources or needs which could be spiritual (personal interests, hobbies, likes/dislikes).
3. Documentation on MHC’s spiritual resources or needs included (use of key words relating to spirituality).
4. MHC unable to provide information due to psychosis or other factors relating to their mental state.

### Focus Group

Staff working in the five MH services included in the study, were invited to participate in a one-hour online focus group. Interested staff members were provided with information about the study and written consent was obtained from all those who participated. A brief overview of the audit’s findings was presented to the focus group participants. Participants were then invited to comment on whether they thought spirituality was addressed during the admission period, and factors which may influence whether this occurred (see appendix 1 for focus group questions). The groups were conducted by the second author who worked externally to the participating MH services.

Brief demographic details were collected (age group, gender, health care discipline, and years of experience). The focus group was recorded and transcribed.

A reflexive thematic analysis (Braun & Clarke, 2021) of the focus group data was conducted inductively, according to the original steps proposed by Braun and Clarke (2006): familiarisation with the data; generating initial codes; searching for themes; reviewing themes; defining and naming themes; and producing the report. The first author reviewed the transcript, generated initial codes using line-by-line analysis, and searched for themes. These themes were then reviewed and refined with the second author (MB), and defined, named and written up into a report. Both authors are experienced qualitative researchers with clinical experience. The philosophical stance taken in this paper was one of critical realism (McEvoy & Richards, 2006).

#### Patient and Public Involvement

As this study specifically focuses on the role of staff in identifying and documenting spiritual resources and needs of MHCs, there was no consumer involvement in the design of this particular study. The study design was developed from findings of the researchers’ recent studies with other healthcare staff, spiritual care specialists, and healthcare recipients.

## Results

### Audit

A total of 205 MHC medical records were audited. See Table 1 for demographic details.

**Table 1:** Audit results, demographic details (*n* = 205)

Variable	*N* (%)
*Gender*	
Male	114 (55.6)
Female	87 (42.4)
Transgender or other	4 (2.0)
*Age Group*	
Under 20	7 (3.4)
20–29	38 (18.5)
30–39	51 (24.9)
40–49	35 (17.1)
50–59	21 (10.2)
60–69	18 (8.8)
70–79	13 (6.3)
80–89	18 (8.8)
90–99	4 (2.0)
*Servic*e	
Safe Assessment Space	32 (15.6)
Psychiatric Emergency Care Centre	40 (19.5)
Inpatient Adult Mental Health	59 (28.8)
Community Mental Health	33 (16.1)
Older People’s Mental Health	41 (20.0)
*Religion (recorded)*	
None	102 (49.8)
Unknown/Not recorded	39 (19.0)
Christian	19 (9.3)
Catholic	15 (7.3)
Anglican	11 (5.4)
Indigenous/Aboriginal *Spirituality*	6 (3.0)
Hinduism	2 (1.0)
Judaism	2 (1.0)
Agnostic	2 (1.0)
Buddhism	1 (0.5)
Mormon	1 (0.5)
Orthodox Christian	1 (0.5)
Protestant	1 (0.5)
Islam	1 (0.5)
Other	2 (1.0)

Table 2 indicates how the records were grouped across the four categories.

**Table 2:** Content of admission documentation

Category	N (%)	Example of entry in medical record
1. No documentation about spiritual resources or needs	84 (41.0)	“Social: private rental, lives by herself, has been working full-time, not currently working” (Caritas)
		“Personal history not explored” (Caritas)
		“Currently lives with Mum. Nil contact with kids. On DSP. Previously worked as tow truck driver and security guard” (SAS)
2. Some documentation about resources or needs which could be spiritual	56 (27.3)	“Enjoys going to the library. Enjoys the computer. Currently writing a book” (OPMH)
		“meditation, nature, Lego/creativity, listen to music, Instagram” (Consumer Wellness Plan – “Things I do well”) (PECC)
		“Utilised IPAD to listen to music and self-soothe” (SAS)
		“Poor interaction until discussion turned to art on his IPAD. MHC became reactive and engaging” (PECC)
3. Documentation on MHC’s spiritual resources or needs identified	17 (8.3)	“Has not left the unit yet and wants to see trees and be outside. Would like to go sit outside under a tree and write in her diary…to take leave this afternoon to get fresh air” (PECC)
		“Attends Evangelist Church at (suburb). Attends weekly on Sundays. Considers himself spiritual” (Caritas)
		“Felt he had a spiritual awakening on return from overseas. Felt increased sense of faith while in Italy, symbolic connections” (IMH)
		MHC recorded to say: “I felt very alive, very accomplished…and was reading online the work of a life counsellor with a Buddhist orientation” (CMH)
		“is spending her day by engaging in some volunteer duties such as delivering program leaflets to co-residents and attended chapel/church services. These activities provide her sense of purpose and comfort” (OPMH)
4. MHC unable to provide information due to psychosis or other factors relating to their mental state	49 (23.9)	“Dismissive of any meaningful engagement” (IMH)
		“Story hard to follow” (IMH)
		“very difficult to get a clear story of recent events due to tangentiality and over circumstantiality” (SAS)

Most of the information recorded during the admission period aligned with the first category. This information was often collected on an assessment form (known as an A1) or summarised in consultations with doctors or nurses. Information about a MHC’s hobbies and interests (second category) were occasionally recorded on the A1, in the doctor’s notes, or included in nursing observations. In the PECC unit a “Consumer Wellness Plan” was completed on admission, and included such information. Data was included in the third category if clear questions about a person’s spiritual or religious history were asked or there was documentation pertaining to a MHC’s spiritual resources or needs. Several MHCs were diagnosed with acute psychosis, which impacted upon any meaningful interactions (fourth category).

### Focus group

Six MHPs participated in the online focus groups (see Table 3 for demographic details).

**Table 3:** Focus group participants (*n* = 6)

Variable	*N* (%)
*Gender*	
Female	6 (100.0)
Male	0 (0.0)
*Age*	
20–29	1
30–39	2
40–49	1
50–59	1
60–69	1
*Role*	
Social worker	2
Nurse manager	1
Nurse	1
Psychology	1
Medical	1
Years of Experience, M (SD)	11.7 (10.0)
*Ancestry*	
British/Irish	2
British	1
British/Australian	1
Nepalese	1
Chinese/Australian/Indonesian	1
*Religion*	
None	3
Hinduism	1
Catholic	1
*Anglican*	1
*Religious person*	
Strongly disagree	1
Disagree	2
Neither agree nor disagree	3
Agree	0
Strongly agree	0
*Spiritual person*	
Strongly disagree	1
Disagree	1
Neither agree nor disagree	0
Agree	2
Strongly agree	2

The analysis of focus group data identified four over-arching themes (see below). The data from the audit and focus group is represented in an integrated format in Table 4.

Theme 1) What information about MHCs’ spiritual resources and needs is currently collected and when

**Table 4:** Integrated data across audit and focus group

Audit category	Relevant focus group content
1. No documentation about spiritual resources or needs	Spirituality is often associated with religion
	Asking about spiritual resources or needs may incite new trauma
	Asking about meaning and purpose may be awkward if they have none
2. Some documentation about resources or needs which could be spiritual	Spirituality is best identified over time through relationship
	Asking about a MHCs daily routines and interests can reveal more about them that may be meaningful
3. Documentation on MHC’s spiritual resources or needs identified	Spirituality can be a protective factor against suicide
	Facilitating a MHC’s spiritual resources may help identify places or activities where they feel calm
	Spirituality may be better addressed with staff training and more comprehensive care planning
4. MHC unable to provide information due to psychosis or other factors relating to their mental state	Knowing about a person’s spirituality may help establish whether they are well or experiencing psychosis

During the focus group discussion, it became apparent that most of the participants associated the word “spirituality” with religious faith or beliefs. When asked whether spirituality would be included in their initial assessment, one participant responded: *“I would have said no because I think when you say spirituality, my lens goes to like faith and like religion”* (Doctor)*.* When a broader definition of spirituality was provided by the researchers, participants agreed that they probably did include references to MHCs’ spiritual resources and needs but would not use that language. A distinction was created between information collected at: i) initial assessment, and ii) over the treatment period.

1. Initial assessment.
  According to participants, spiritual resources or needs were rarely identified or documented during the admission period. Instead, much of the focus was on assessing risk to self or others; understanding the reason for presentation; and identifying protective factors. A manager commented, “*that's the thing that I probably look for is the risk. The risk associated with the presentation”.* A doctor spoke about her role admitting psychiatric MHCs to hospital. For her, the main questions at admission were: *“why is this person presenting with this diagnosis, and what am I going to do about that?”.* Details would then be documented in a “formulation”, which could include protective factors: “*…if something’s particularly relevant I will mention it* [a protective factor] *there* [in the formulation]* …if you know someone has any protective factor that that's going to be a good thing kind of prognostically”.*

Another participant agreed and reported that during an initial assessment she would look for protective factors: “*So sometime when we do initial assessment, we talk about what do you like to do on your free time or what kind of activities make you feel relaxed or like happy, joyful. In that case, some people they like to ‘I walk my dog’. ‘I go walking*’” (Nurse). Although this information might potentially include spiritual factors, the focus was more upon hobbies or interests rather than aspects that might connect them to something of ultimate meaning or significance.

2. Over the treatment period.
  Participants described how information collected at admission could be distinguished from the amount and depth of information collected over time. This was noted by community MHPs. A Social Worker spoke of how visiting a person’s home was one way to gather further information and cues about their spiritual resources. She commented on a recent home-visit with a MHC: “*He's a very old person. He's probably not going to live for that long, but there's a way in which that provided some sort of meaning to him and, connection to his life*” (Social Worker 1).

Getting to know a MHC’s usual routines and activities also helped staff to better identify some of their spiritual resources: *“I often ask people about their routines, their daily routines, what kind of places they go, and people will bring up church…and that can be a good kind of base point for asking about what kind of support does the church give you?”* (Neuropsychologist).

According to several participants, the spiritual resources and needs of MHCs were often discussed within a team but not documented in the MHC medical record. This could happen at gatherings such as the “morning huddle” or at a weekly case conference: “*these things don't get documented, but there is an exchange of information”* (Social Worker 2).

Theme 2) Perceived benefits of knowing about MHC spirituality

Once they were aware of the broader definition of spirituality, participants were able to articulate four benefits of knowing about MHC spirituality. First, such knowledge was perceived to help them engage with MHCs and provide support. For instance, another Social Worker spoke of how the knowledge of a MHC’s religious beliefs prompted her to take the MHC to the hospital chapel, which subsequently helped the MHC to feel calmer. She went on to explain: “*being able to sort of do that in a way that's very conversational …can be a way to elicit a whole lot of information that's really helpful and can give you some insights into what might be meaningful for somebody and give them purpose”* (Social Worker 1)*.*

Second, such information could be incorporated into a plan at discharge to help prevent relapse: *“protective factors and spirituality probably comes into it around that time… what are we doing again in that period of time to keep someone well.”* (Doctor). A third benefit proposed by participants was that some beliefs (usually religious) could act as a protective factor specifically in relation to suicide risk. One participant described how “*when I’m doing a suicide risk assessment and I’m asking what stops you from acting on that? What keeps you going?”* there were a few MHCs who had told her “*right at the forefront it’s my Catholic faith…it’s against my faith, I believe that someone is looking out for me, and that’s come up quite early in the assessment*” (Neuropsychologist).

Last, knowing about a MHC’s spiritual beliefs could help staff gauge whether MHC’s beliefs were psychotic, or part of their usual system of beliefs.

*I once saw a young gentleman who had this sort of conviction that he needed to fly overseas and go on a mission and spread the word of God, and in some cultural backgrounds, or religious backgrounds obviously that's very normal, but …this was actually very out of character for this young man. So, in that situation, although he was very psychotic in the end, it took a bit of time to work out like is this real or not?* (Doctor)

Theme 3) Reasons why spirituality is not documented by staff in the medical record.

There were several reasons participants would not enquire about a MHC’s spiritual resources and needs: a MHC might be experiencing a MH crisis because of a lack of source of meaning or purpose in their lives; a MHC may have experienced trauma related to religious or spiritual beliefs in the past; or talk of religion or spirituality might exacerbate a MHC’s psychosis.

Participants reported that they were reluctant to explore spiritual resources or needs of MHCs when there was an evident lack of purpose or meaning in their life already. Either there “*was not much to comment on*” (Doctor), or such exploration may take the conversation along a negative path.

*My worry is asking a question like “what meaning do you have in your life?” is that a lot of our MHCs don't have something that brings them meaning…it just seems like the kind of question that…really kind of heads down a bit more of a negative avenue* (Neuropsychologist).

It was also thought that talk of religion with some MHCs may be stressful due to traumatic experiences. One participant explained:

*I'm reluctant to bring it up because I don't know what someone's experience has been with faith and spirituality. And while a lot of the times it's positive actually, the opposite is true for a lot of people. It's a very traumatic experience and so I don't want to incite new trauma by bringing it up in a review* (Doctor).

Lastly, participants mentioned that talk of religion or spirituality may exacerbate psychosis for some MHCs due to their strong delusional beliefs:

*If I'm working … and there's a MHC that presents who's psychotic I am probably not at that point gonna explore things that might exacerbate his psychotic symptoms. And like often…the psychotic symptoms, can be about things like religion and spiritual things. So I guess that would be when I would really probably not explore spirituality* (Manager).

In addition to the impact upon the MHC, there were two other issues relating to staff members’ reluctance to discuss spiritual issues with MHCs. The first reason was the perceived shortfalls of existing assessment tools. The A1, it was explained, did not specifically include spiritual resources or needs and had little space to for personal details. Another participant felt that exploring spiritual resources and needs was better suited to a more conversational discussion where one could…* “elicit a whole lot of information that that's really helpful and can give you some insights into what might be meaningful for somebody”* (Social Worker 1).

Lack of time was a second factor. This varied according to the discipline of the participant, with the doctor having the least amount of time:

*But if you have seven people waiting in ED … I probably then only have half an hour with someone to make a decision about whether they need to stay in hospital or whether they need to go…a lot of the time, you just don't get time to talk about that detail*.

Theme 4) What would help staff better document spiritual resources and needs of MHCs?

Two areas of potential change were identified. The first was to improve staff awareness of spiritual issues. One participant described how, by working alongside Aboriginal Australians, she had developed a greater sensitivity to spiritual and cultural matters for MHCs. An example of this changing awareness was appreciating the importance of connection to country that many Aboriginal Australians experience, and understanding the disruption to this connection during hospitalisation.

*An example of that might be somebody who has left that country, which might be somewhere in the Outback and come to this busy city and is now struggling to cope and whereas I might not have picked up on that connection to country in the past it's something that I now would* (Manager).

Another spoke of how her own personal spiritual experiences helped her to be more mindful of the spiritual resources and needs of her MHCs. It was suggested that staff training to develop knowledge and awareness around spiritual resources and needs could be introduced.

The second suggestion for promoting better incorporation of MHCs’ spiritual needs was through care planning. A care plan could be added to over time and represented important aspects of the MHC journey. One participant had used care plans in another country and found that a care plan was a useful document where spiritual resources and needs of the MHC could be included: *“…they were very individual to the clients and they were made really once we've gotten to know them. So … any kind of spiritual or religious needs would be adopted into that care plan”* (Manager).

## Discussion

The aim of this study was to investigate how the spiritual resources and needs of MHCs are currently documented in the initial assessment period, and to explore how MHPs identify the spiritual resources and needs of MHCs in their care. The findings of the medical record audit revealed that most information collected during the initial admission period was limited to factors such as medical and social history. Approximately one quarter of records additionally included information on a consumer’s interests or hobbies, and a much smaller proportion (less than 10%) specifically included information relating to spiritual resources or needs. The focus group data provided further explanation of this data, with staff relaying how the spiritual resources were more likely to be identified with MHCs over time and as a relationship with staff developed. Furthermore, although benefits were identified, there were clear reasons why staff would not ask a MHC about spirituality early in an admission. To assist staff to better enquire about the spiritual resources and needs of MHCs over time, it was suggested that staff awareness of spiritual issues be enhanced, and care planning be implemented.

Our participants were concerned that enquiry about a MHC’s spiritual resources or needs may, in some situations, worsen the MHC’s condition. Koenig et al. (2020) have outlined clinical guidelines for MHPs in relation to spiritual assessment, such as: how to take a spiritual history; supporting the MHC’s beliefs; encouraging religious participation; participating in religious activity; and referral to clergy. They also acknowledge the difficulties which may arise when religion may be part of the problem for a MHC, such as when a religious struggle may be a source of distress or be a manifestation of their psychosis. In such situations they advocate a gentle approach be adopted by the clinician, where the MHC is listened to and supported.

One of the findings of this study was that staff felt that greater awareness of spiritual issues would assist them to provide better support to MHCs. This is a common finding across health (Jones et al., 2020b, 2021a, 2021b; Klitzman, 2021). Unlike the Joint Commission of Accreditation of Healthcare Organisations (JCAHO) in the USA, Australia’s regulatory body (the Australian Health Service Safety and Quality Accreditation, AHSSQA) does not place an emphasis on the collection of a patient’s spiritual information. Little teaching in spiritual care is provided in Australian medical curricula (Wenham et al., 2021). The focus group participants revealed some of their discomfort around discussing spiritual issues citing awkwardness when a MHC appeared to have no meaning in their life, *“it just seems like the kind of question that…heads down a bit more of a negative avenue”.* Discomfort with discussing spirituality has been identified as a barrier to spiritual care in previous research (Best et al., 2016). It is difficult to separate discomfort from lack of training, as spiritual care education is known to provide improved confidence and competence in exploring spiritual issues with patients (Jones et al., 2024a, 2024b).

Spiritual care training programs have been implemented in the area of MH to build knowledge, confidence and competency of MHCs in discussing spiritual care, and have been demonstrated to be effective. For example, three studies have examined the development and effectiveness of an eight-module online program for MHPs (Pearce et al., 2019, 2020; Salcone et al., 2023). The program was demonstrated to improve the confidence and competence of MHPs in providing spiritual care (Pearce et al., 2020), and these findings were replicated recently (Salcone et al., 2023). Similar findings have arisen as a result of spiritual care training in other healthcare contexts (Jones et al., 2020a, 2021a, 2021b). Research has demonstrated that staff who view themselves as spiritual or religious are more likely to provide spiritual care to MHCs (Neathery et al., 2020).

This study emphasised the importance of the relationship between MHCs and MHPs. According to focus group participants, spirituality was more likely to be discussed with MHCs when a trusting relationship had been developed over time. This preference for a more relational exchange of information rather than formal assessment, has been noted in other Australian research (Best, 2023; Jones et al., 2021a, 2021b). Shortfalls of assessment tools were identified by focus group participants, and the benefits of a more conversational approach over time were highlighted. These findings are consistent with models of care delivery in mental health settings that also emphasise the importance of a safe and trusting relationship between MHPs and MHCs (Barker & Buchanan-Barker, 2010). With increased awareness and enhanced sensitivity regarding the spiritual and cultural needs of Aboriginal Australians who access the healthcare system (McBride et al., 2021; NSW Ministry of Health, 2020), the value of relationships and listening rather than taking a tick-box approach, is necessary.

One of the recommendations by the focus group in this study was for services to introduce better care planning across both inpatient and community settings. The benefits of collaborative care planning in MH have been documented (Coffey et al., 2019). These benefits are seen when MHPs adopt patient-centred care, and consumers are meaningfully and actively involved in care-planning (Coffey et al., 2019; Scott & Aboud, 2021). However challenges of care planning have also been identified (Scott & Aboud, 2021). In a systematic review, Scott and Aboud found that tokenism and scepticism from MHPs are significant barrier to good care planning. Furthermore, the time involved in data collection can be perceived as time-consuming and arduous (Scott & Aboud, 2021). To address these issues the authors encourage mental health services to prioritise the “relevance of care planning to the everyday lives” of MHCs, and avoid yielding to bureaucratic requirements that may place stifle such relevance.

### Limitations

This study was limited in several ways. An audit can only identify what has been written. Participants revealed that information is often discussed verbally and not recorded in MHC files, and they also identified problems with the current assessment forms. The focus group sample was small, and although representative of several disciplines, contained members of one gender only. Religious and ethnic diversity was also limited. Although there was potential to include a younger cohort in this study (there is a younger person’s mental health service at St Vincent’s), it was beyond the scope of this study to compare such a group with older adults. This may be worthy of consideration for future projects, as young Australians have demonstrated an interest in spirituality (McCrindle & Renton, 2021).

## Conclusion

It was clear in this study that spiritual needs and resources of MHCs are rarely documented at time of admission to a MH service. On further exploration with staff, it was discovered that there are key reasons why staff do not discuss spiritual needs or resources with MHCs at this early stage, preferring to do so over time. However, this may result in many spiritual resources and needs being missed at a time they are most important. Better staff education and care planning may provide avenues for staff to be better equipped to gently address this aspect of a MHC’s life earlier in the healthcare journey.

## Focus Group Interview Protocol

1. Describe the information that you collect in an initial assessment when a MHC is admitted to your service?
2. Spirituality has been defined broadly as an “aspect of humanity that refers to the way individuals seek and express meaning and purpose and the way they experience their connectedness to the moment, to self, to others, to nature, and to the significant or sacred”. With this definition in mind, is spirituality something you feel is routinely addressed during an initial assessment?
  - If so, what are some of the questions you or other staff may ask a MHC upon their initial admission to your service?
  - If not, why do you think this is the case?
3. What factors might impact upon whether spirituality is covered during an initial assessment?
4. Are there particular challenges or circumstances that may make it difficult to ask a MHC about spirituality?
5. What might help you to ask a MHC about spirituality during an initial assessment?
6. Thank you for your time. Before we finish, is there anything else you would like to mention about documenting the spiritual resources of MHCs during an initial assessment?
